# A direct comparison of real time PCR on plasma and blood to detect *Plasmodium falciparum* infection in children

**DOI:** 10.1186/1475-2875-11-201

**Published:** 2012-06-15

**Authors:** Abigail A Lamikanra, Carlota Dobaño, Alfons Jiménez, Augusto Nhabomba, Hoi P Tsang, Caterina Guinovart, Maria N Manaca, Llorenç Quinto, Ruth Aguilar, Pau Cisteró, Pedro L Alonso, David J Roberts, Alfredo Mayor

**Affiliations:** 1 Nuffield Department of Clinical Laboratory Sciences, University of Oxford, Headington, Oxford, OX3 9DU, UK; 2 National Health Service Blood and Transplant, John Radcliffe Hospital, Headington, Oxford, OX3 9BQ, UK; 3 Barcelona Center for International Health Research (CRESIB), Hospital Clínic-Universitat de Barcelona, Rosselló, 132, 4th floor, Barcelona, 08036, Spain; 4 Centro de Investigação em Saúde da Manhiça (CISM), Vila de Manhiça, Maputo, CP 1929, Mozambique

**Keywords:** Malaria, qPCR, Plasma, Anaemia

## Abstract

**Background:**

Estimation of *Plasmodium falciparum* parasitaemia can vary with the method used and time of sampling. Quantitative real time PCR (qPCR) on whole blood or plasma samples has previously been shown to be more sensitive than thick film microscopy. However the efficiencies of each method have not been compared using samples obtained from infants less than one year old.

**Methods:**

A multiple of statistical approaches were used to compare the performance of qPCR on whole blood or plasma to detect the 18 S ribosomal gene of *P. falciparum* in 548 samples from children aged 2.5 or 24 months. Parasite prevalence in matched samples was compared using Mcnemar’s test and agreement of positive results quantified as Kappa scores. Parasite prevalences between different age groups were compared by Fisher’s test. Results from analyses by thick film microscopy were also available from children at 24 months and their correlation to each qPCR method examined by the Spearman’s test. Finally the association of *P. falciparum* infection with the incidence of multiple malaria episodes from contact to 24 months of age was evaluated using negative binomial regression.

**Results:**

These analyses showed that qPCR from whole blood detected approximately 3-fold more cases of infection than plasma qPCR. Both qPCR methods agreed well with each other although qPCR from plasma had a greater agreement with microscopy (96.85%) than did qPCR from blood (69.7%). At 24 months the prevalence of infection detected by all methods was associated with anaemia (p < 0.05).

**Conclusions:**

The data presented here demonstrates that low levels of parasitaemia are better detected by qPCR using parasite DNA from whole blood than from plasma. However plasma samples provide a viable substitute when parasite smears are unavailable.

## Background

*Plasmodium falciparum* malaria continues to affect over 200 million people and accounts for over half a million deaths mostly among children [1]. Whilst rapid treatment of malaria cases with effective anti-malarials, the use of insecticide-treated nets and new combined therapies have recently helped to significantly reduce the mortality associated with malaria, there is still a risk that emergence of resistance to these approaches will occur. The ability to monitor relative levels of parasitaemia is, therefore, of paramount importance to reduce malaria morbidity and mortality and to control and eradicate malaria, as well as to evaluate clinical trials [2].

Previous work has shown that PCR of archived plasma samples can be used to detect *P. falciparum* malaria [3]. In that study PCR from plasma and blood were more sensitive than microscopy at detecting parasitaemia during the early onset of disease. However, performance of PCR on blood and PCR on plasma were not compared. More recently other reports have demonstrated that quantitative PCR (qPCR) on DNA extracted from blood can be used to differentiate between species of Plasmodium [4] and can be a more sensitive method to quantify parasitaemia than microscopy of thick smears [5,6].

Here the performance of qPCR on frozen plasma samples was compared with qPCR on whole blood collected onto filter paper to detect and quantify relative parasite levels in young children from Mozambique. This was with the aim of providing information on the relative utility of plasma samples where whole blood or blood smears are unavailable. Both whole blood and plasma samples were available from participants at 2.5 (n = 293) and 24 months of age (n = 255) for qPCR assessment of parasitaemia. Blood smears at 24 months were also available for evaluation of parasitaemia by microscopy.

## Methods

### Study area

The study was conducted at the Centro de Investigação em Saúde de Manhiça (CISM), located in Manhiça, Maputo Province, southern Mozambique. There, transmission of *P. falciparum* is perennial with marked seasonality and of moderate intensity, with an entomological inoculation rate of approximately 40 infective bites/person/year [7].

### Study design and procedures

The study was conducted in the context of a double-blind randomized placebo-controlled trial (NCT00231452) carried out in Manhiça from September 2005 to March 2009 [8]. HIV-negative pregnant women were recruited during the third trimester of pregnancy at the antenatal clinic and their children received chemoprophylaxis with sulphadoxine-pyrimethamine (SP) plus artesunate (AS) or placebo during predefined periods of their first year of life. Study participants were followed up until age 24 months. At 2.5 and 24 months, blood was collected by finger-prick to measure haemoglobin and to spot 50 μl onto filter paper no. 903TM; Schleicher & Schuell. One mL of blood was also collected into EDTA microtainers, centrifuged and plasma stored at −80°C. At 24 months, blood smears were also collected.

Weekly active case detection was conducted from birth to age 10.5 months and monthly home visits from 10.5 to 24 months. Additionally, passive case detection was carried out through the morbidity surveillance system to monitor attendances to the outpatient clinics and admissions to hospital. Parasitaemia and haematocrit were determined in children with fever (auxiliary temperature ≥37.5°C) or whose guardians reported history of fever in the preceding 24 h. If the slide was positive for asexual *Plasmodium* parasitaemia, they were treated with SP or oral quinine if children had received a study intervention within the previous 2 weeks.

Informed consent from pregnant women was sought to enrol their newborn in the study upon birth. The protocol was approved by the National Mozambican Ethics Review Committee, the Hospital Clinic of Barcelona Ethics Review Committee and the National Health Service Oxfordshire Regional Ethical Committee.

### Laboratory methods

Blood slides were read to quantify parasitaemia. Briefly, thick and thin Giemsa-stained films were examined using a light microscope and oil immersion lens. Slides were declared negative only after 2,000 leucocytes were counted. All slides were read by two different microscopists and a third read was performed if the ratio of densities from the first 2 readings was >1.5. Haematocrit was measured in microcapillary tubes with a Hawksley haematocrit reader after centrifugation.

Plasma samples were stored at −80°C for 3 to 4 years before extraction of DNA. Dried blood spots collected onto filter paper were stored for the same period at 4°C with silica gel. DNA was extracted from a 50 μl spot of blood on filter papers with QIAamp DNA Blood Mini Kit and from 50 μl of plasma with QIAamp DNAeasy Blood & Tissue Kit (both from QIAGEN) and finally resuspended in 100μl water and 30μl AE buffer, respectively.

DNA extracted from blood and plasma was amplified in an ABI PRISM 7900 HT Real-Time System (Applied Biosystems) following a previously described qPCR method [9] that is specific for the 18 S ribosomal RNA gene of *P. falciparum* (accession number M19173). Briefly, qPCR on samples from blood was performed in a 20 μl reaction containing 5 μl of template in 1x TaqMan® Universal qPCR Master Mix (Applied Biosystems), 300 nM of each primer (Fwd: GTA ATT GGA ATG ATA GGA ATT TAC AAG GT 3’, Tm 59°C; Rev: 5’ TCA ACT ACG AAC GTT TTA ACT GCA AC 3’, Tm 61°C) and 150 nM of TaqMan probe: 5’- FAM - TGC CAG CAG CCG CGG TAA TTC TAMRA-3’. Reactions using DNA from plasma were in 25 μl with 300nM of each primer (Fwd: 5’-GCT TTT GAG AGG TTT TGT TAC TTT GAG-3’, Tm 61°C; Rev: 5’-TTC CAT GCT GTA GTA TTC AAA CAC AAT-3’, Tm 60°C) and 200nM of TaqMan probe: (5’- FAM- AAG GTG AAC GTG GAT GAA GTT GGT GG-TAMRA-3’) (all from Applied Biosystems). Amplification and detection were performed under the following conditions: 2 min at 50°C, 10 min at 95°C, and 40 cycles (45 cycles for plasma) of 15 s at 95°C and 1 min at 60°C. Each sample was run at least in duplicate and a negative-control sample with no template DNA was included in all plates. Results were analysed using ABI Prism software (Applied Biosystems). Each of these methods was standardized in 2 different laboratories.

### Definitions and statistical methods

Anaemia was defined as haemoglobin <8 g/dL and a clinical malaria episode as *P. falciparum* asexual stage parasites of any density observed by microscopy plus axillary temperature ≥ 37.5°C or history of fever during the previous 24 h. The prevalence of *P. falciparum* positive samples by qPCR from blood and matched plasma were compared by McNemar´s test. Agreement of positive results between techniques was quantified as Kappa score. Prevalence was compared between age groups by Fisher’s test. The Spearman’s test was used to determine the correlation between microscopy and qPCR cycle threshold (C_T_) values. Negative binomial regression was used to evaluate the associations of the *P. falciparum* infections with the incidence of multiple malaria episodes from contact to 24 months of age.

## Results

A total of 548 samples were analysed by qPCR on DNA extracted from whole blood on filter paper (qPCR-blood) and plasma (qPCR-plasma). Of these, 143 (26%) were found to be *P. falciparum* positive by qPCR-blood. qPCR-plasma detected 37 (7%) as positive. Agreement between both techniques was 78.1%, with a Kappa score of 0.253 (Standard deviation [SD] 0.032). Seven of the positive samples detected using plasma-DNA were negative when blood-DNA was analysed. Conversely 113 samples that were identified as positive using blood-DNA were negative using plasma-DNA.

Samples from a total of 307 children were analysed for this study and 241 of these were available at both 2.5 and 24 months. Of these, 16 samples were positive at both time points using qPCR-blood and 2 were positive using qPCR-plasma. An additional 52 samples were examined from children at 2.5 months and 14 samples from children at 24 months. Therefore in total 293 were collected from children at 2.5 months of age and 255 from children aged 24 months (Table 1). Children in the younger age group had a lower prevalence of infection than older children (p < 0.001) when detected using qPCR-blood or qPCR-plasma.

**Table 1:** **Comparison of qPCR methods used to determine the prevalence of*P. falciparum*infection in children aged 2.5 months and 24 months**

		**2.5 months**	**24 months**	
		**n = 293**	**n = 255**	p*
**Pf infection, n (%)**				
	**qPCR Blood**	43 (15)	100 (39)	<0.001
	**qPCR Plasma**	8 (3)	29 (11)	<0.001
	**Microscopy**	ND	32 (12.5)	
**C**_**T**_**value, Median (IQR)**				
	**qPCR blood**	38.0 (36.9-38.5)	36.8 (32.0-38.1)	<0.001
	**qPCR plasma**	40.7 (38.3-41.6)	37.9 (33.8-41.0)	0.285
**Anaemia, n (%)**		5 (2)	23 (9)	<0.001

* Comparison between each time point by fisher’s test (prevalences) and Kruskall Walis test (C_T_ value).

*ND*, not determined, *IQR* Interquartile range.

Data from microscopy was available from 24-month old children. The C_T_ values from both qPCR methods correlated significantly with parasite densities by microscopy (p < 0.001) where qPCR-plasma gave a Spearman’s rank correlation coefficient (rho) value of −0.753 and qPCR-blood a rho value of −0.823. Parasite infection detected by microscopy showed greater agreement with detection by qPCR-plasma (96.85%) than did qPCR-blood (69.7%) with Kappa (SD) scores of 0.844 (0.063) and 0.275 (0.047) respectively.

Table 2 presents the association between anaemia and the prevalence of *P. falciparum* infection. The proportion of children with anaemia was higher among infected than uninfected children, especially those at 24 months of age. At 24 months of age, the incidence rate of having had a previous malaria infection was higher among children positive by qPCR-blood (incidence rate ratio [IRR] 3.50, 95% CI[2.04-6.30], p < 0.001), qPCR-plasma (IRR 3.55, 95% CI[1.56-8.10], p = 0.003) or microscopy (IRR 3.92, 95% CI[1.83-8.40], p < 0.001), compared to negative children (analysis adjusted by use of insecticide treated nets, intra-door residual spraying and treatment).

**Table 2:** **The association between the prevalence of anaemia in children and*P. falciparum*detected using 3 different methods**

**Age**	**Method**	**Pf infection, n**		**Anaemia, n (%)**		
**(months)**				**Yes**	**No**	p*
**2.5**	**qPCR-blood**	positive	43	1 (2)	42 (98)	0.550
		negative	250	4 (2)	246 (98)	
	**qPCR-plasma**	positive	8	2 (25)	6 (75)	**0.006**
		negative	285	3 (1)	282 (99)	
**24**	**qPCR-blood**	positive	100	14 (14)	86 (86)	**0.042**
		negative	154	9 (6)	145 (94)	
	**qPCR-plasma**	positive	29	7 (24)	22 (76)	**0.008**
		negative	225	16 (7)	209 (93)	
	**Microscopy**	positive	32	8 (25)	24 (75)	**0.002**
		negative	252	15 (6)	237 (94)	

* Fisher’s tests. Pf, *P. falciparum.*

## Discussion

Here the rate of detecting *P. falciparum* by qPCR using DNA isolated from plasma and from whole blood in children during a longitudinal study conducted in Mozambique was compared. There was agreement between both qPCR methods in both younger (2.5 months) and older (24 months) children, where the detection of parasitaemia by qPCR from blood and plasma was associated with higher prevalence of anaemia. However, qPCR-blood detected parasitaemia in approximately 3.5 fold more samples than qPCR-plasma or thick film microscopy. Accordingly, qPCR-plasma showed greater agreement with microscopy than qPCR-blood.

The qPCR primers and probes for each assay targeted different DNA sequences within the same gene of *P. falciparum*. Although each method was optimized this may explain some of the observed differences in detection. The greater detection rate using blood as a source of parasite DNA could also be attributed to less parasite DNA in plasma since whole blood would contain more parasitized RBCs. The C_T_ values obtained from both plasma and blood taken at 2.5 months were 1 to 2 cycles greater than values obtained at 24 months. This suggests parasite burden would have been 2 fold less in younger children which could accentuate the differences in detection rate between each qPCR method at 2.5 months.

The choice of primers to target *P. falciparum* in this study is supported by recent work that demonstrates targeting the 18 S ribosomal RNA gene is more sensitive than targeting mitochondrial cytochrome b [10]. These authors also used glycogen/acetate precipitation to improve yields of parasite DNA. Others have shown that measurement of both parasite RNA and DNA by qPCR can improve sensitivity and detection of almost 40% more positive cases [11]. Use of these approaches would improve detection rates with the qPCR methods described here. Primers targeting other Plasmodium species may also be of use in detecting sub-microscopic infections by non-falciparum parasites, especially in areas where they may be prevalent. Studies are ongoing to assess the prevalence of sub-microscopic infections with other Plasmodium species in the Manhiça study area.

In this study parasite burden measured by all methods was associated with prevalence of anaemia at 24 months. Moreover, children with *P. falciparum* infection detected by any of the methods used (PCR on plasma, PCR on whole blood or microscopy) were at higher risk of having had a previous malaria episode. However the higher detection rate using qPCR-blood and its association with greater prevalence of anaemia supports the importance of detecting sub-microscopic malaria infection to improve diagnosis and management of infections in children under five years of age [12,13].

The density and range of *P. falciparum* infection also influences protective immunity to *P. falciparum*[14]. Malaria interventions would therefore benefit from use of accurate and sensitive methods to monitor parasite prevalence in study participants. Knowledge of which samples to collect, when they can be best utilized and the relationship between measured parasitaemia using different methods will help to inform studies in which measurement of immune correlates is required.

In many clinics located in malaria endemic countries, the use of real time qPCR is too expensive and slow compared with use of microscopy. However the data presented here supports the use of molecular methods to improve surveillance and treatment of malaria. In areas where the prevalence of infection is considerably low a recently described method of pooling samples could decrease the number of reactions required and therefore reduce the costs and time associated with using qPCR [15]. This approach, together with developments in the use of loop-mediated isothermal amplification [16], may assist in making molecular detection of malaria infection in the field more common.

## Conclusions

To conclude, qPCR using parasite DNA from whole blood is more sensitive to detect sub-microscopic levels of parasitaemia than using parasite DNA from plasma. However, the data presented here demonstrates that the performance of qPCR on plasma samples is similar to the performance of microscopy, suggesting that qPCR on plasma can be used as a substitute to microscopy when performing retrospective studies with limited material and when blood smears are unavailable.

## Abbreviations

qPCR, Quantitative real time PCR; CT, Cycle threshold.

## Competing interests

The authors declare that they have no competing interests.

## Authors’ contributions

AAL contributed to acquisition, interpretation of data, drafting and revision of the manuscript, CD, AJ, AN, CG, MM, LQ and RA contributed to acquisition of the data, HPT and PC contributed to acquisition and analysis of data, PA critically revised and gave approval of the version to be published DJR Contributed to the conception, interpretation and design of the study, has critically revised and given approval of the version to be published, AM Contributed to the conception, interpretation and design of the study, drafting and revision of the manuscript, and given approval of the version to be published. All authors have read and approved the final manuscript.
